# From embracement to attachment: Registering people on the move and the couplings of noncitizenship and territory

**DOI:** 10.1177/23996544251322768

**Published:** 2025-02-24

**Authors:** Paul Trauttmansdorff, Annalisa Pelizza

**Affiliations:** 9184 Technical University of Munich, Germany; 9296 University of Bologna, Italy; 1006 University of Århus, Denmark

**Keywords:** Registration infrastructures, border databases, (non)citizenship, territories, attachment

## Abstract

This article analyzes how people on the move encounter registration infrastructures on their journey to Europe. Through the lens of three empirical cases—registration in the Eurodac system, registration in the framework of the European temporary protection directive, and national registration of migrants on Hellenic islands—we explore what we term “noncitizenship-territory couplings.” The article develops the concept of “registration as attachment” to describe how sociotechnical procedures establish relationships between noncitizen subjects, mobility rights, and territorial space. We therefore ask how registration infrastructures couple territory and noncitizenship in Europe in different ways—that is, how such couplings shape the movement of people, enact territories, and endow spatial rights or restrictions. We argue that new and partial couplings of noncitizenship and territory are produced—the “containment coupling,” the “facilitation coupling,” and the “detainment coupling.” Each follows specific registration dynamics, enacts different territorial patterns, and simultaneously shapes migrant mobility and their rights. By situating the article at the intersection of science and technology studies, human geography, and citizenship studies, we wish to contribute to the growing literature on the performative effects of infrastructures in border and migration governance. At the same time, our intervention encourages further analysis of the varied spatialities of states and sovereign power that move beyond current discussions about denationalization and deterritorialization.

## Introduction

Information infrastructures have key roles in shaping the movement of people. They can prevent, facilitate, or block movement; they shape the categories of belonging and difference, which classify mobile populations into subjects with different status, rights, and obligations. Recent literature at the intersection of critical migration studies, border studies, and science and technology studies (STS) has called for the analysis of how infrastructures are entangled with the production of borders and migrant (im)mobilities. This call refers to the study of the myriad infrastructural processes, techniques, and tactics of registration and categorization and asks how these processes organize mobilities across space and time (Amelung et al., 2020; Dijstelbloem, 2021; Leese et al., 2022; Lin et al., 2017; Pelizza, 2020; Trauttmansdorff, 2024).

Following this call, our article asks how mobile people encounter infrastructures at different sites of registration in Europe. Scholars have illustrated in many ways how these encounters subject people to various digital and physical bordering procedures, numerous relationships with authorities and non-state actors, and multiple institutional orders (De Goede, 2017; Kuster and Tsianos, 2016; Pollozek and Passoth, 2019). We thus broadly consider *registration infrastructures* as a particularly important entry point into the study of today’s highly differentiated border and mobility regimes.

As the encounter between people and authorities, the result of which is formally recorded, registration consists of the articulation of multiple forms of work: the production, negotiation and processing of data; the coordination of actors, organization, and databases; as well as the performance of identity (of both individuals and polities). In current European migration governance, registration often constitutes the first moment in which third-country nationals still unknown to European authorities are “coupled” with access rights to territory (Sassen, 2006). And yet, unlike the modernist coupling of citizens and territory that characterizes an individual’s belonging to the nation-state, contemporary registration of people on the move creates links to spaces that are more complex and partial than this traditional understanding of national belonging.

Our article analyzes three distinct registration infrastructures in Europe: The Eurodac/Dublin infrastructure, the infrastructure of temporary protection for Ukrainian refugees, and the Hellenic border infrastructures using the Register of Foreigners (HRF). Each of these infrastructures comprises constellations of actors, databases, and data practices, along with their associated political-legal frameworks. Notably, they exemplify diverse ways in which mobile people become “attached” to information systems, that is, translated from potentially unknown individuals into digitally recordable, processable, and “European-legible” identities (Pelizza 2020). Registration infrastructures, in terms of Collier and Ong (2003), inscribe “the space and form of limited, finite, and localizable relationships and effects that occupy a certain space and that concretely link—or distinguish and divide—various objects, spaces, techniques, individuals, and social groups” (p. 423). Collier and Ong’s definition invites us to explore the *spatial effects* of registration, that is, the relationships between individuals on the move and the space they wish to traverse or are allowed to cross and inhabit. The latter also corresponds to what Nira Yuval-Davis (2006) calls their *spatial rights*, that is, “the right to enter a state or any other territory of a political community and, once inside, the right to remain there” (p. 208). Therefore, we ask how registration infrastructures couple territory and noncitizenship in Europe, and how these couplings shape the movement of people, enact modes of access, and endow specific spatial rights or restrictions.

More precisely, through three cases, we explore manifestations of what we call “noncitizenship-territory couplings.” Registration infrastructures, we argue, establish different couplings than the one defined by the traditional modern contract, enforced by the nation-state and linking an individual’s exclusive belonging to national territory. Instead, registration infrastructures establish new relationships between mobile people and territories. Different from most analyses emphasizing deterritorialization, our article seeks to describe *novel, partial couplings* that follow various registration dynamics in response to human movement, enact different territories, and link them to human subjects and their rights. In doing so, we aim to contribute to the infrastructure-oriented studies of mobility and border regimes. However, we also use this lens to encourage further analysis into the spatialities of states and sovereign power beyond discussions of denationalization and deterritorialization at the intersection of human geography, citizenship studies, and STS.

In the following sections, we review some of the important strands in this multifaceted and interdisciplinary literature. This will allow us to refine our idea of noncitizenship-territory couplings and the concept of registration as attachment and justify our article’s emphasis on infrastructures. Subsequently, we provide an overview of our empirical material and methodological approach. Finally, we analyze our three cases of registration infrastructures in Europe.

## The decoupling of noncitizenship and territory

### Deterritorializing space and governance

Conventional understandings of citizenship and territory are tied closely to the emergence of the modern national state. The ideal type of the state in the modern period, with the monopoly of violence and the centralized production capital and coercion as the foundation of sovereignty (Tilly, 1990: 21), defined citizenry in relation to an individual who belonged to a bounded territory. It created a form of political space distinct to the modern state, as Walters remarks, invoking “a certain symmetry of governance and geographical area” (Walters, 2009, 488). This national state model also emerged from competition with other forms of sovereignty. In feudalist systems, political authority was built on personalized bonds that cemented rigid social hierarchies (Strayer, 1965); city-states created their own jurisdictions and sovereign economies in which citizen-classes were granted partial or differentiated rights (Braudel, 1985: 479–558). The national state model, in turn, implied that substantial control over people was enforced by binding them to a geographically homogeneous, isotropic, and contiguous area, as Farinelli (2009) describes modern territory. The state would then also impose a common language, a single religion, or a uniform currency; it would use extensive cartographic techniques and forms of knowledge production that enacted homogeneous space, translating people and land into national population and territory (Branch, 2013; Möllers, 2021; Mukerji, 2011; Scott, 1998).

John Torpey has highlighted the essential role of documentary registration and identification in the production of states—what he called the historical process of embracement: “[T]he activities by which states ‘embrace’ populations were essential to the production and reproduction of states in the modern period” (1998, 245).^1^ Embracement expropriated mobile people from the rights and means to move (just like workers were expropriated from the means of production in industrial capitalism). It articulated the state rulers’ desire to enact individuals as distinct members of a population on national territory. The process of embracement determined who belonged to this political space, drew geographical boundaries, and endowed rights, entitlements and obligations to both the ruled population and “foreigners” (which usually underwent closer surveillance and restriction). In other words, the governing state *coupled* citizenship with national-territorial space.

Since Agnew’s essay on the “territorial trap” (1994), scholarship in human geography, political sociology, and border and migration studies have problematized this nation-state-centered understanding of space and its coupling with citizenship, questioning the reification of territorial space as a prefixed unit of sovereignty. Global assemblages and their denationalized geographies of capital, technologies, authority and rights, have been at the center of the work of scholars like Sassen (2006) or Mezzadra and Neilson (2013). Processes of de- and reterritorialization have been propelled by surveillance and control technologies and led to the proliferation of state borders beyond, and deep within, national territories (see Amoore, 2006; Paasi, 1998; Papadopoulos et al., 2008; Walters, 2002). The core of these arguments concerns the rejection of the notion of territory as a bounded given, as an absolute or homogenous geography. Instead, as geographers such as Painter and Allen claim, scholarship should begin considering territory as a *performed* category, “not so much a source of power as one of its possible effects” (Allen, 2011, 287; Painter, 2010).

### Deterritorializing belonging and citizenship

Likewise, citizenship studies scholars have discussed the deterritorialization of citizenship—perceived conventionally as *de jure* relationship between the state and the citizen. Accordingly, scholars have claimed that citizenship can no longer be grounded exclusively in the territoriality of the national state, which articulated the citizen’s formal status of belonging to the nation. Instead, citizenship has increasingly been studied as de facto category with hybrid and deterritorialized elements, for instance, “plugged into transnational networks of markets, technology, and expertise” (Ong, 2006, 499). Isin and Neilson’s proposal to study “acts of citizenship” (2008) as practices of doing (i.e., not receiving) citizenship emphasized the possibility of enacting belonging and subjectivity in multiple ways. Soysal’s account of the guestworker experiences in the European Union is a prominent case to illustrate a post-national condition of belonging “[in] a time when national citizenship is losing ground to a more universal model of membership, anchored in deterritorialized notions of persons’ rights” (1994, 3). Studies adopting the perspective of deterritorialization have occasionally coined concepts of citizenship as a flexible (Ong, 1999) or relational arrangement (Pols, 2016; M’charek and Casartelli, 2019), as a fluid and dynamic conception (Isin, 2009), as a matter of affection and anxiety (Fortier, 2016), or as a fragmented category (Esposito et al., 2020).

Scholarship stressing dynamics of deterritorialization therefore addresses the heterogeneous ways of understanding and doing citizenship not in juridical or ontological terms, but as an outcome especially of *practices of othering*. The partial blindness of more conventional citizenship concepts would stem from a formalistic expression of belonging as state membership: “while cast in the language of inclusion, belonging and universalism, modern citizenship has systematically made certain groups strangers and outsiders” (Isin and Turner, 2002, 3). Tambakaki connects to these findings when she proposes to attend to *noncitizenship*, an umbrella term that describes “the exclusions, inequalities, injustices and naturalizations that accompany citizenship politics” (2015, 923). In her understanding, marginalized groups, such as those commonly defined as migrants or refugees, actively “contest and challenge the inside–outside distinction that the state system perpetuates and […] embody a type of citizenship that exists alongside standard accounts” (p. 926). It is through their mobility and activities at the fringes of the formal state system that noncitizens challenge established juridical dimensions of citizenship (Esposito et al., 2020; Nyers, 2003).^2^

All in all, both literature strands on deterritorialization and noncitizenship have been successful in problematizing the traditional understanding of the coupling of citizenship and territory, which cannot be taken as a univocal benchmark of existing forms of belonging and rights. Drawing on those, we propose to further highlight the performative potential of noncitizenship to articulate more complex couplings than the one invoked by the formal status of national belonging. Our focus on registration infrastructures can then demonstrate how *novel, partial couplings between territories and noncitizenship* emerge. In the next section, we will present a theoretical approach based on the notion we introduce as “attachment,” which will help us further explain these couplings.

## Infrastructural registration as attachment

STS and critical border studies have often drawn on the above literature to disentangle how infrastructures establish, consolidate, or create new categories of noncitizenship. Amelung, Gianolla, Solovova, and Ribeiro contend, for instance, that categories “are de jure and de facto entangled in such a way that the absence of a formal citizen status implies new forms of misrecognition, restriction or lack of human rights, thus othering and discriminating people” (2020, 587). Pelizza and Van Rossem (2023) have provided a typology of “scripts of alterity,” stressing the agency of registration infrastructures in enacting, and not only representing, people on the move as othered. Furthermore, while producing and constraining noncitizenship, infrastructures also shape patterns of mobility and access to territories (Dijstelbloem, 2021).

This STS literature has also built upon a long-standing tradition in the history of technology and state formation of critiquing the naturalized conception of the territorial state. Scholars have emphasized that the modern imaginary of the state as a demarcated territorial unit has been constructed and continually performed by modern technological, infrastructural, and scientific methods and practices (Carroll, 2006; Mitchell, 2002; Möllers, 2021; Mukerji, 2011; Passoth and Rowland, 2010). An infrastructural lens has thereby helped to review (state) borders as provisional, incomplete patchworks that require vast amounts of harmonization and standardization work (Leese, 2018; Tazzioli and Walters, 2016). Likewise, it illustrates how territorial and citizenship orders are partial and contingent once the symbolic representations of territory and citizenship as exclusive properties of the state are no longer taken for granted. Drawing on this understanding will help us illustrate how registration infrastructures establish novel and partial couplings of noncitizenship and territory.

While, for the reasons discussed above, we seek to move away from the exclusive paradigm of embracement and belonging, we suggest instead exploring the processes of registration through what we refer to as “attachment.” The term attachment has previously been employed in the context of studying markets and consumers (Callon and Law, 2005) or big data and data subjects (Ruppert et al., 2015). In our context, we relate *attachment* to the procedures, IT systems, activities, interactions, and transactions through which mobile people become *database subjects*. For our analysis, we see registration in a database as an attachment in order *to acknowledge that (mobile) individuals are bound to specific territorial arrangements only qua enactments as specific database subjects*. In other words, registration leads to the production of subjects that do not exist as isolated, independent entities but are enacted through their mutual relationship with territory. People attached to a database can, for example, cross regional boundaries but not national ones; or, alternatively, their mobility can be restricted to sub-regional and local areas.

The idea of attachment therefore adopts a pragmatist understanding of subjects as outcomes of acts of registration. With this formulation, we also take into account the lesson of human geographers in understanding territory as the performed effect of power, considering the proliferation of borders beyond and within national territories. Attachments thus go against the view that territory is conceived merely as a geographical space, a naturalized and bounded object of rule. By contrast, they allow us to apprehend space as a *relational notion* rather than an Euclidean metric (Allen, 2011, 289; Grommé and Ruppert, 2020; Paasi, 2011). Such an approach considers multiple spatial relationships as potential outcomes, with different power effects and radically diverging experiences of nearness and distance. Attachment then means registering an individual within a virtual space—a database, as well as onto a geographical space—a geometrically variable territory.

Moreover, registration as attachment takes into account citizenship studies’ lesson to differentiate between different formations and modalities of citizenship. We propose to extend the idea of embracement as the process of establishing national “belonging,” that is, “identifying persons unambiguously in order to control their movements and to distinguish members from nonmembers” (Torpey, 1998: 239). Using attachment as a concept instead allows us to grasp the more complex relationships between subjects, authorities and space. Describing registration into a database as a materially bounded and reversible process, attachment is much closer to the idea of “being plugged in” than of “belonging” (Ong, 2006). It establishes a relationship not simply with a state, but with the offices that run and have access to databases (such as Eurodac’s “Dublin Unit” typically hosted at interior ministries). As scholarship has shown, such authorities are more fragmented than the assumption of a discrete, coherent state would suggest (Pelizza and Loschi, 2023; Pettrachin and Pelizza, 2024). While embracement concerns the expropriation of the rights and means of movement, attachment articulates the (partial) right to move in more complex geometries. More specifically, while embracement determines who belongs to the homogeneously imagined political space of the state, attachment establishes who can access a non-homogeneous, discontinuous space that is nested within and across states. Yet attachment does not necessarily assume an exclusively deterritorialized understanding of mobility rights. As the act of registration in a database, it instead enacts mobile individuals as subjects that can be bound to different territorial geometries. In other words, the realization of such geometries as non-homogeneous does not entail a lack of territory but lack of a homogeneous one. It is this complex production of variable geometries and non-homogeneous territories that our article seeks to map in the remaining sections.

## Notes on methodology

Our empirical material for this paper has been collected during the six-year long project *Processing Citizenship*. We believe that, as a multi-scalar polity, Europe constitutes an important case in point to study the dynamic couplings of noncitizenship and territory. Here, the institutional architecture of modern state jurisdiction is complemented with supranational bodies that operate in complex ways, (re-)distributing essential functions, tasks, and responsibilities across national and supranational actors. It is this multi-scalar, sociotechnical architecture that has been the core focus of *Processing Citizenship* and provides the background for our interest in emerging couplings of noncitizenship and territories.

Over the course of the Project, data collection was accomplished by triangulating ethnographic fieldwork, interviews, and policy, technical or administrative documents.^3^ For this specific contribution, the authors mainly drew on ethnographic observation, interviews with refugees, operators of reception centers, registration officials and police authorities, and technical and administrative documents collected in reception and identification centers in Italy in the summer of 2017, as well as in Greece from March to October 2018. As the project unfolded, new phenomena at multiple sites came to light—war, unprecedented patterns of (im)mobilities, and new forms of registration—opening pathways for deeper engagement with *comparative frameworks*. We thus, moreover, started to analyze and compare documents and reports from fieldwork in arrival centers in Austria during March and April 2023, where interviews with officials, police authorities, agency representatives, or NGO staff were conducted.^4^

For the first two cases of this article, we provide selected field observations as starting points to elaborate on the role of registration as attachment. Their analysis is complemented with interview material and documents that reflect the politico-legal context of the cases. Text passages were analyzed mostly through thematic and in-vivo coding until two main abstract themes were constructed and their relationship theorized and verified: “enacted (database) subject” and “territorial pattern.” Drawing on the framework above, the relationship of these themes was then analyzed more in-depth and served as the core focus for the comparison across the three cases.

Our first case, the Eurodac/Dublin infrastructure, builds on observational material that has previously been published to present an argument centered on identification and translation (see Pelizza, 2021). The material is now mobilized to move a different argument, and it is complemented with the analysis of policy, technical documents, and interviews collected during fieldwork in Italy/Greece, which focused on police and asylum registrations of people crossing the Schengen borders without the required authorization. Eurodac, the European dactyloscopy database that stores fingerprints of asylum seekers and so-called “irregular migrants,” constitutes the main component of what we term the Eurodac/Dublin infrastructure. Its goal is to contain migrants and asylum seekers and their movements via the Dublin III Regulation (EU, 2013), regulating—and indeed preventing—secondary movements or so-called practices of “asylum-shopping,” that is, asylum applications lodged in at least two EU Schengen countries.

Our second case, the infrastructure of temporary protection, focuses on the arrival and reception infrastructures for displaced persons from Ukraine who are protected by the European Council Decision 2022/382. Shortly after the Russian invasion of Ukraine, millions of Ukrainian residents crossed the Schengen border with European neighboring countries, leading to more than 5.9 million Ukrainian people in Europe.^5^ Our case focuses on Austria, the fourth biggest receiving member state, where approximately 450,000 travelers entered, becoming directed to arrival centers established in cities such as Vienna, Graz, or Linz.^6^ We will detail the registration infrastructure at one of Vienna’s main locations, the so-called First Arrival Center Althanstraße, jointly hosted by the Red Cross, Caritas, and Austrian police authorities.

Our third case, the bordering infrastructure relying on the Hellenic Register of Foreigners (HRF), focuses on the registration of refugees on Hellenic islands. With this case, we illustrate the practices of myriad actors in the attachment process which produces both “vulnerable” and “nonvulnerable” subjects within reception centers. Here, we center specifically on the enactment of insular territories for detaining mobility—yet another lens through which we attempt to describe how territorial arrangements and forms of noncitizenship become coupled.

By distinguishing these cases as separate infrastructures, they allow us to adopt a comparative lens that highlights their performative qualities and governmental rationales. Comparison contains a specific analytical vantage point that foregrounds how meaning emerges through difference (Van Rossem and Pelizza, 2022). Ours is therefore a deliberate case construction that serves a methodological purpose, namely, to step back and reflect the effects of performing registration. It aims to create comparable parameters while avoiding crude generalizations. To this end, our analysis emphasizes the situated character of registration and tries to foreground highly contingent attachments, both making visible how territory and noncitizenship become coupled differently and creating the sensation that “things could be otherwise.”

## The containment coupling: Enacting suspicious irregulars and nested territories

Our first case zooms into registration within the Eurodac/Dublin infrastructure. Eurodac, the European biometric system for storing fingerprints of asylum seekers and irregular migrants (with plans to include facial scans soon), has consistently been the central database of this infrastructure. It has established fingerprinting as the primary technique of gathering information in the European governance of asylum. Under the Dublin III framework (EU, 2013), EU member states are obliged to record and transfer, and exchange fingerprints and other data and to either “take back” or “take charge” of asylum seekers for the purposes of processing claims for international protection. The input and collection of bodily data occur when migrants and refugees are apprehended within the Schengen territory, cross Schengen borders, or seek asylum. The core rationale, as Lewkowicz specifies, has been to “turn the non-European body into a readable document, fixing bureaucratic identities onto bodies and rendering precarious photographic representation obsolete” (2023, 6). Consequently, a mobile individual who is registered within this infrastructure, traced and subsequently identified through a “search hit,” may be subject to deportation based on the legal obligations of state authorities to take back individuals “who made an application in another Member State or who is on the territory of another Member State without a residence document” (EU, 2013, Art. 18).

The process of registration at a center in mainland Greece is usually carried out as a series of interlinked steps of attachment. To illustrate this case, we use a field observation from Pelizza (2021, 489–503) in a registration center in Greece.

Registration starts with photos taken in a dedicated modular building. During photo making, officers are supported by a photocopied hand-written form reporting categories like surname, name and date of birth. Officers hand-write in the values (i.e. actual name, date of birth etc.).

[…]

After [some] preparation, the officer places the persons’ hands on the scanner surface and runs the digitizing software by selecting the ‘Eurodac’ tab in the Hellenic Register of Foreigners. The digitizing software is provided by the European Commission and is integrated with the Hellenic Register only at the interface level: the Hellenic and the European systems do not share data pools. […]

Sometimes [scanned] images [of fingerprints] are not of good quality, and then the officer repeats the scanning. In some cases, even the second scanning does not solve the problem, as fingerprints are worn. If image quality is sufficient, they are uploaded. The officer, like most of his colleagues, is not sure where they are uploaded and stored. After a few interviews, we understand they are initially uploaded on the Hellenic Criminal Agency database. From there, they are uploaded on Eurodac, the European system for asylum. To do that, images are automatically converted into bit-strings, a requirement posed by the European Parliament to implement privacy by design. Once uploaded on Eurodac, bit-strings allow searches to check whether an individual has already applied for asylum in another country (hit/no-hit). If a match is found, contextual information about the person is returned (e.g., reason for previous fingerprint collection, country of first entry, etc.) and asylum officers at the Centre will signal the case to the Member State where the asylum request was first lodged.

This scenario of registration involves multiple interconnected components such as various actors, technologies, documents, interrogation methods, and standards. At every stage of this procedure, there is a potential for disruption, corruption, or hindrance. It is only when the individual’s fingerprint finally lands in the central repository of Eurodac, becoming a unique token of their mobile identity, that the *attachment* is complete. Attachment to Eurodac enrolls the individual on the move as a *suspicious and irregular subject* (see also Pelizza and Van Rossem, 2023), and above all it enacts a specific territorial space within which the subject’s movement should be contained.

Our definition of the coupling is formulated, first, by examining *how* attachment enacts people on the move as subjects within Eurodac, and second, by specifying the territory to which *access* is granted to the database subjects. Primarily, attachment is here characterized by the suspicion performed by authorities during the registration process. Eurodac registrations specifically focus on *when* and *where* a mobile individual is apprehended and documented, simultaneously seeking to substantiate their asylum claims while subjecting them to suspicion. Eurodac registrations scrutinize various aspects of people’s bodily representation, their country of origin, the authenticity of their documents, the credibility of travel routes, or the conduct of the registering officer in performing their national obligation. This scrutiny encompasses information like the date when the fingerprints were captured, the date on which data are transmitted to the central system, when the person left the territory of a member state, or the date when the person was removed from member state territory.

Suspicion could, therefore, be seen as an integral element of the Eurodac/Dublin infrastructure and its attempt to govern migrant movement. The objectives of its underlying regulatory framework, the Dublin III agreement, institutionalize the assumption of irregularity in the movement of third-country nationals. They focus on preventing secondary movements, multiple applications of asylum, or what is derogatorily termed asylum-shopping.^7^ Suspicion plays a key role in the concerns of national authorities, particularly regarding the subject’s “illegal” stay on a state’s territory and its so-called “risk of absconding” (EU, 2013, Art. 28). Acting on Dublin III, authorities must appear to have legitimate reasons “to believe that an applicant or a third-country national or a stateless person […] may abscond” (Art. 2).

Attachment within the Eurodac/Dublin infrastructure, therefore, implies *containing* movement within Europe to national boundaries. While applicants must wait for a response to their asylum request, they are only allowed to move within the member state in which their request was lodged. While existing literature connects this containment to the temporal micromanagement of people on the move—for Tazzioli and Garelli, for example, it disrupts and decelerates migrant geographies (Tazzioli and Garelli, 2020, 1012), we deem it necessary to linger on the spatial dimension, and specifically on how containment enacts specific territories. Europe is here not enacted as a homogenous space nor a centralized sovereign entity. On the contrary, the containment of mobility through the Eurodac/Dublin infrastructure represents governing asylum in a territorially fragmented and decentralized manner. For Eurodac subjects, the European space does not emerge as a transnationally harmonized area but rather as a series of “nested territories.” Asylum applicants find themselves confined to these sub-spaces, that is, the national space of the responsible member state, *nested* within the broader European Schengen region. The Eurodac/Dublin infrastructure thus establishes a definite coupling between noncitizenship and territory, which we refer to as the “containment coupling.” Territoriality is here produced as a series of nested spaces, simultaneously to the enacted subject as a *suspicious irregular*. Eurodac achieves this by creating digital traces of migrants’ fingerprints, effectively marking their bodies as a border, and interconnecting asylum authorities, police stations, and refugee hotspots across the continent. At the same time, this infrastructure reterritorializes national spaces and obliges national authorities to limit the movements of applicants within their national boundaries.

## The facilitation coupling: Enacting transient European citizens and homogeneous territory

Our second case documents the registration of individuals who were fleeing the Russian invasion of Ukraine. This registration was mandated by EU Decision 2022/382, which activated the Temporary Protection Directive (TPD) to grant Ukrainian citizens protection and the freedom to move within the EU Schengen area. The TPD put in place “minimum standards for giving temporary protection in the event of mass influx of displaced persons,” facing a “high migratory pressure” on its Eastern borders due to the unfolding war (EU, 2022: 1).^8^ Furthermore, the European Commission (EC) issued “operational guidelines” that emphasized the importance of registering personal data of refugees (EC, 2022; EU, 2001, Art. 10/Annex II), such as name, nationality, date and place of birth, marital status, and family relationship. At the same time, the EC encouraged European member state authorities to introduce measures to facilitate the orderly entry of “mass influx” of forced refugees from Ukraine as temporary beneficiaries of protection.^9,^^10^

Our field observation zooms into the registration procedures taking place at the “Althanstraße First Arrival Center” in Vienna in March 2023, which served as the primary reception facility. Following the outbreak of the war, most travelers arriving in Austria were directed to arrival centers strategically established in cities like Vienna, Graz, or Linz. These checkpoints were intended to manage arrivals by guiding them to various social services, like “steering customers” to consultations provided by NGOs like the Red Cross, Diakonie, or Caritas (Interview with city official, March 2023). They offered assistance for private or organized accommodation and access to public employment services, local authorities, or COVID-19 testing. Subsequently, police authorities became involved in these centers by setting up a central registration infrastructure.

At the reception desk of the Althanstraße facility, an initial step of “in-house registration” typically takes place. Red Cross staff record basic personal information, including name, date of birth, gender, and day of check-in. Subsequently, colored wristbands are distributed to visitors in order to identify individuals within the building and differentiate between those seeking accommodation and those undergoing official registration—which is officially known as “Erfassung” to denote the requirement for a permanent residence in Austria, access benefits, or obtain work permission.

At Althanstraße, a police officer offers a brief tour of the facility and the rooms where approximately 40 registrations are carried out daily. The officer emphasizes that the registration process is typically straightforward and uncomplicated, often taking no more than five minutes. However, during our visit, the registration of a Ukrainian mother and her daughter highlights some of the well-known challenges typical of similar contexts. When entering identity information, the Austrian police utilizes GREKO-FAM, which is an old national registration interface previously employed at border-crossing points. The initial step involves scanning a Ukrainian passport, which typically adheres to international biometric standards, including a chip with photo and essential personal information. While possessing an international passport is usually a legal requirement for travel, we are told that many displaced individuals from Ukraine can complete formal registration without one (for example with a domestic passport or a national identity card). In such cases, additional evidence of a Schengen border crossing is necessary, acting as a substitute for the stamp typically found in a passport.

In a subsequent step, a three-page document known as the “Erfassungsblatt,” the data collection sheet, is photocopied and filed. It contains personal information, an Austrian address, particulars about citizenship status, travel history, date of entry, and any criminal record. Then, registration involves capturing 10 fingerprints and digitally inputting them into the system—a practically challenging step that can require multiple attempts. At this point, we leave our observation because once this fingerprint data becomes processed, additional actors take over registration. In particular, the data sets are examined in the back offices of the Federal Office for Immigration and Asylum. For applicants, this part of the process remains opaque. It may result in no outcome at all, with only positive assessments leading to the issuance of the so-called “Blue Card,” the ID for displaced people, which should be delivered to the person’s official address. Negative results are not communicated and result in individuals being excluded from applying for basic care and social benefits.

In the fieldnote above, a complex process involving technological artefacts, using interfaces, issuing documents, filling forms, performing evaluations, and producing declaration cards, must ensure that the individual becomes attached to the main national information system called GVS/BIS.^11^ GVS/BIS operates as the Austrian database for persons in need for “basic care,” centrally run by the Ministry of Interior and connected with regional authorities. The database contains a wide range of personal information—name, address, country of origin, residency status, date of entry, received services from the state, etc., and is accessible to ministry officials, the Federal Agency for Reception and Support Services, as well as local authorities. Through this *attachment*, people attain the status of a member of the “target group,” aligning with the implementation of the European TPD. Local authorities and city officials subsequently work with the data set in the GVS/BIS database. Here, a “client’s” basic care ID, known as the “GVS number,” is generated, with which access to a range of basic social benefits can be sought.^12^ For Ukrainian refugees, the possession of a “Blue Card” confirms their attachment to the GVS/BIS system and their status as displaced beneficiaries. The positive assessment by authorities essentially completes the attachment: registration translates an officially unknown mobile individual into an officially recognized and temporarily protected beneficiary.

In this scenario, attachment enacts a subject that we propose to call the *transient European citizen.* Unlike Eurodac registrations, suspicion does not play a central role in the formation of these subjects. Fingerprints, for instance, do not hold significant importance to authorities and serve as a “mere reassurance” (Interview with national agency official, March 2023). As one official explained, there was “across civil service administration, across all the ministries, across federal states, […] general willingness to help in unbureaucratic manners, something you would hardly expect as a trained Austrian [state official]” (Interview with national agency official, March 2023). The authorities tended to overlook lost or forgotten identity documents, and any missing documents were not turned into “problems” for registration.

Consequently, registration into this national data space enacted a transient European citizen with a harmonized set of minimal social rights and benefits. This attachment could, however, also prove to be fragile, as demonstrated by several bureaucratic intricacies. For example, state authorities gradually began rejecting requests for basic assistance from individuals who had arrived in Schengen before February 24, 2022—the official date marking the onset of the war. Numerous individuals who had fled before the outbreak suddenly found themselves unauthorized in Austria, classified as “illegal immigrants.” As one police officer recalled: “If you held a residence permit, let’s say, in Slovakia, dated on 27^th^ of January, you are illegally present in Austria and, in theory, subject to punishment” (Interview with police officer, March 2023). To alleviate these situations, authorities often recommended returning to Ukraine for a brief period of time, and then re-entering with the “correct” passport stamp.^13^ Becoming registered with this stamp, in combination with a valid Ukrainian passport, remained the crucial passage point for becoming a transient European citizen.^14^

It should be noted that attachment to this national data space enables access to what is invoked as a homogeneous Schengen territory. One of the primary goals of European governments in offering protection was the necessity to handle “mass influx” without engaging authorities in the resource-intensive and protracted procedures of asylum (including long-term benefits of granted asylums). A city official, when reflecting on the temporary protection directive, summarized the key objective of enabling Ukrainian refugees to freely enter the Schengen spaces: “they actually need[ed] to move quickly, wherever they want[ed], and not get caught up at any borders” (Interview with city official, March 2023). This perspective is further supported by the EC’s “operational guidelines” (EC, 2022) issued shortly after the adoption of the TPD. They framed Eastern Schengen borders as chokepoints requiring the “temporary relaxation of border controls” (p. 3). Contrary to other third-country nationals, the EC suggested facilitation measures, “flexibility” or the “simplification” of controls “for certain categories including vulnerable persons,” “special arrangements,” or “waiving of customs duties and measures to facilitate the entry of pet animals travelling with their owners” (pp. 1–2).

The rationale pursued by European governments is clearly one of facilitating movement, which enacts Ukrainian refugees as transient European citizens knowledgeable of a *Schengen space enacted as homogeneous and accessible territory*. Particularly in the initial period following the onset of the war, this rationale aimed to channel displaced people to destinations of their choice, where they could unite with family relatives or contacts already residing in the European Union (Interview with Austrian NGO, March 2023). Countries like Austria or Germany supported this effort with free public transportation and assisting travel between Ukraine and the preferred destination within Europe.^15^

The infrastructure of temporary protection illustrates the enactment of a transient European citizen who receives a minimum of protection from the national welfare state (qua attachment to the national data space), and, concurrently, enjoys the right to move throughout Europe. Attachment thus couples a specific temporary citizenship to a European territory—with “privileges” categorically denied to those enacted as irregular immigrants.^16^ The *temporary* nature of this facilitation coupling is crucial: the right to access, encompassing both national social benefits and the European Schengen territory, is granted only for a one-year duration according to the TPD, with the potential of annual extensions up to three consecutive years, contingent on renewed decision-making by the Council. This situation presents beneficiaries with what one of our interlocutors labels the “waiting dilemma” (Interview with national agency official, March 2023). Transient citizens find themselves at a crossroads, often torn between returning to a war-afflicted home or building a new life in Europe. Enacted as transient subjects, however, they are also “protected” from, and restrained in, imagining a longer-term future. The infrastructure of temporary protection fosters waiting by withholding any prospects of a longer-term future. A temporary citizen is envisioned primarily to be mobile, to be circulated, toward a clear destination on European territory; yet this exclusive right of mobility is confined to a strictly limited timeframe.

## The detainment coupling: Enacting detainees and vulnerable together with insular and nested territories

Our third case focuses on registration on the Hellenic islands, specifically in the facilities known as Hotspots that conducted reception and identification tasks (some of which have been rebranded as “Closed Controlled Access Centers”). The Hellenic Reception and Identification Service (RIS) officially manages these centers, which are intended to accommodate individuals from third countries or stateless people who arrive in Europe and apply for international protection. The registration of populations on archipelagos has a long-standing history of using islands as instruments of control, exercising sovereign state power, and facilitating offshore detention (Mountz, 2011). In the context of European border governance, registration on island territories has been characterized as messy, modifiable, and at times even experimental undertaking, serving crisis management and consolidation (Pallister-Wilkins, 2020). This is due to the ever-evolving arrangements between diverse actors present, involving state and non-state entities, international organizations, EU agency personnel, national law enforcement agencies, social workers, interpreters, and medical professionals.

In our case, we zoom into the initial reception procedure, which involves a series of steps that can seem arbitrary and bewildering to arriving individuals (Interview on Hellenic Island, April 2018). The registration process is facilitated by collaborating actors such as Frontex, the police, Hellenic asylum service authorities (HAS), RIS personnel, or interpreters. The majority of individuals go through an initial reception phase, which typically includes immediate medical screening and assessment, and identification through the Hellenic Register of Foreigners (HRF). Subsequently, those who wish to lodge an asylum request complete a formal asylum application, again through the HRF platform. It is important to note that HRF registrations on islands by the RIS represent an *additional layer of attachment*—beyond what we previously discussed as Eurodac/Dublin registrations (case 1) and has distinct implications.

Specifically, multiple forms and documents are required to consolidate the traveler’s information, typically gathered in a spreadsheet file and then uploaded alongside the traveler’s fingerprint onto the HRF. A pivotal aspect of the attachment here is the medical screening process, during which the individual can provide details about their medical history or health status in an interview setting, and is facilitated by healthcare professionals like doctors, psychologists or social workers from the Centre for Disease Control and Prevention (“KEELPNO”). The information gathered during medical screening is likewise recorded in a spreadsheet file, which, at the time of research, was mainly used internally among staff, while parts of the spreadsheet data were formally uploaded onto the HRF by RIS personnel.

**Figure 1.**
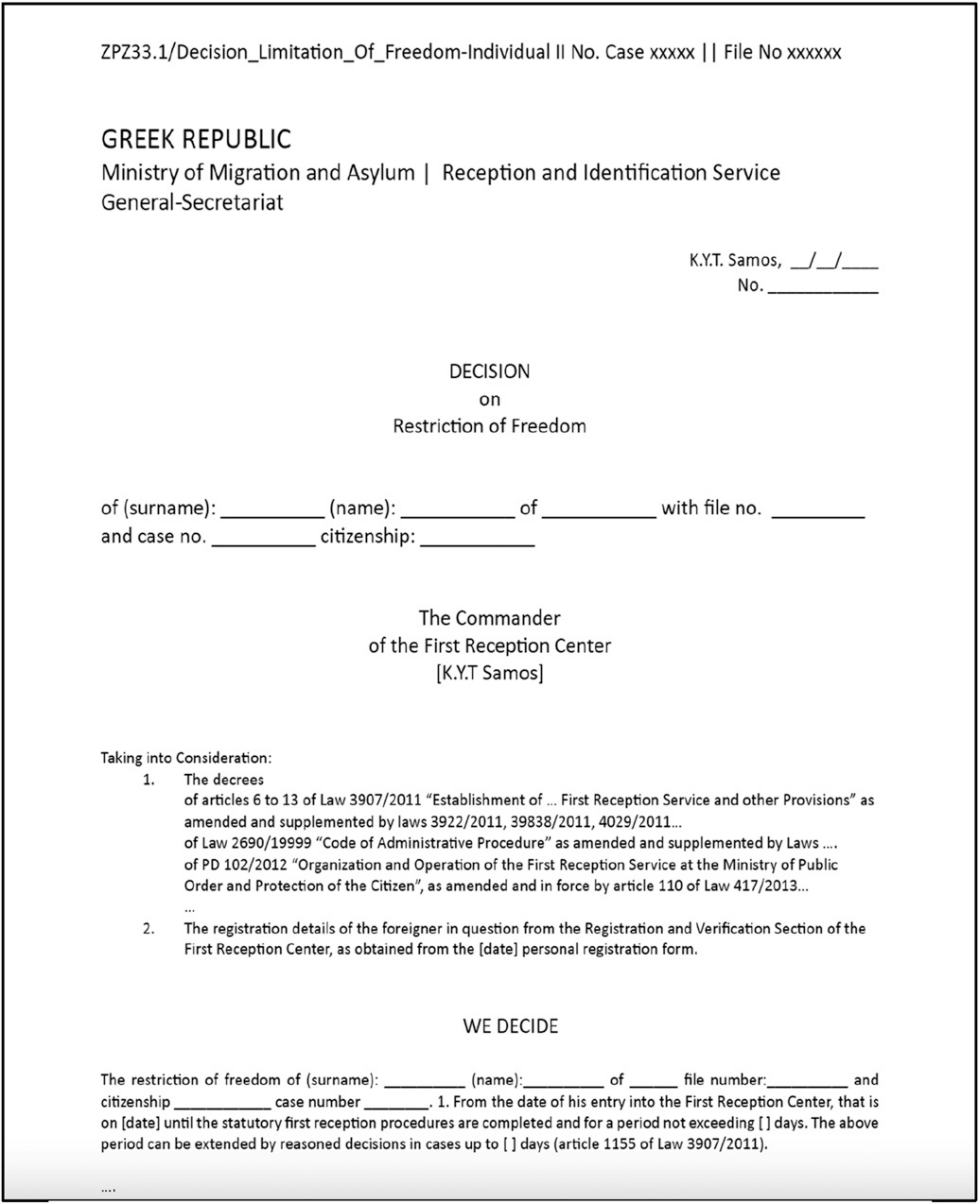
Reconstruction by authors of the official Hellenic declaration of mobility restriction.

Attachment to the HRF leads to critical outcomes, including decisions regarding the restriction of freedom for both families and individual applicants (see Figure 1 for a reconstruction of an official declaration). It also involves the issuance of a “small card” (referred to as “Karthia”), which signifies the individual’s pending asylum status and carries a stamp indicating movement restrictions.^17^ Additionally, a health card may be provided. These artifacts together determine the status of a noncitizen subject within the HRF system on the island. The immediate imposition of movement restrictions is a crucial consequence of this process, and it is especially experienced for individuals within the “Fast-Track Border Procedure” and those affected by the EU-Turkey agreement (see ECRE, 2021). Notably, de facto detention is imposed by the police authorities and the asylum service on individuals who may not yet possess an asylum seeker card, either because it is still pending issuance, or their applications have been rejected.

The process of attaching travelers to the HRF thus leads to significant consequences that depend on medical screening results and the evaluation of a person’s declared vulnerability. Medical screening can often be crucial for providing life-saving care, but it also potentially expands (or limits) a subject’s options to move and travel to mainland Europe. Vulnerable cases receive special attention from the RIS, and applicants with vulnerabilities can be transferred to Athens and the mainland for specialized treatment and further medical assessments (Interview with HAS authorities, 2018). In short, vulnerable cases may obtain documented provisions that exempt them from geographical restrictions. For instance, the area of restriction can be lifted for registered individuals in cases where appropriate support can simply not be provided within the camp.^18^

Attachment to the HRF thus results in enacting two distinct categories of subjects. The first category corresponds to those *suspicious irregular subjects* of the containment coupling (see case 1), but here they are additionally confined to island territory. The second subject is enacted as *vulnerable migrant*, and this category may extend the geographical scope of mobility. Being registered as vulnerable may significantly expand the space they have access to and (at least temporarily) alleviate the condition of detainment. Interviewees expressed genuine relief about having a medical report stamped by a hospital that demonstrated their vulnerability and their extended right to be mobile. At the same time, others expressed frustration, feeling that the “system” arbitrarily rejected their vulnerable status and, consequently, their ability to leave insular territory for Hellenic mainland. In either case, individuals pondered the certified status of vulnerability as a desirable asset to become more mobile (Interview on Hellenic Island, April 2018; Interview on Hellenic Island, August 2018).

The process of registering individuals in the HRF encompasses a combination of exercising mobility control and medical care, involves strategies of detainment and opportunities for escape, ultimately resulting in yet another distinct noncitizenship-territory coupling—the *detainment coupling*. The primary purpose of attachment is here to temporarily interrupt the mobility of individuals, enacting *detainees* whose spatial rights are progressively reduced both within and outside reception centers. This form of noncitizenship is here intertwined with insular territory—the spatial arrangement made of land and water, island and mainland spaces. In this context, the archipelago serves as the scattered and extended European border, intensifying isolation and dislocation of detained border-crossers through implementing varying regimes of restriction. For those subjects enacted as vulnerable migrants, in turn, their “Kartiha” card assumes a pivotal role. They may move away at least from the intense and narrow confines of insular territory and access to mainland Greece becomes possible again. In these latter instances, the national territory is reinstated to contain movement within the nested spaces of Europe—once again enforced by nation-state boundaries.

## Discussion and conclusion

Our analysis reveals different couplings of territory and noncitizenship. Each coupling refers to a different form of attachment, enacting database subjects, access rights and territories. The containment coupling was characterized by the enactment of suspicious irregular subjects contained within nested European territories; the facilitation coupling produced transient European citizens with a temporarily homogenous Schengen space; the detainment coupling enacted detainees together with insular territory unless individuals managed to register as vulnerable.

In our view, the results suggest pursuing further research in at least three ways. First, more work needs to be done to understand the manifold spatial politics shaped by registration practices and technologies, as well as the constitutive role of infrastructures for new political spaces. Most studies in this field have built on the insights of scholars who emphasize the performative effects of databases in making institutions and categories, abstracting “data doubles” from their territorial settings (Haggerty and Ericson, 2000), or enabling the operations of governance away from geographical locations to multiple sites of remote control (Amoore, 2006; Dijstelbloem and Broeders, 2015; Popescu, 2011). Much less work has considered the connections between the production of noncitizenship and the production of space, which ultimately leads to markedly different lived experiences of (European) space and its boundaries for mobile individuals. In this article, we started by refining our understanding of seeing (database) registration merely as an instrument for “embracement” by the state (Torpey, 1998). Instead, our concept of registration as materially bounded attachment enabled a more fine-grained analysis; it moved away from embracement as securing an individual’s belonging to a nation-state and illustrated how space and noncitizenship status can be co-produced in several ways, involving multiple actors and artifacts, and yielding critical consequences for mobile people.

A second contribution concerns the debate about the de- and reterritorialization of political space within the so-called post-national condition. Our paper aligns here with the work of border regime scholars who have outlined the multi-scalar geography of Europe, where “multiple vectors and practices of mobility (internal as well as external, even in illegalized forms) have traversed, constituted, and materially transformed the European space” (Altenried et al., 2018: 293). Databases and registration infrastructure, as scholars have demonstrated, have thereby played a crucial role here: they form the “*flickering* foundations” of the European Schengen area as a controlled space (Bellanova and Glouftios, 2022; Leese and Ugolini, 2024); they shape the imaginaries of datafication and data spaces (Baur, 2023; Trauttmansdorff and Felt, 2023); and they produce multiple European geometries and enact populations (Grommé and Ruppert, 2020). Our article instead highlighted the performative production of territories that diverge from official and hegemonic representations of European space, such as the seamless and harmonized area of Schengen mobility reserved for EU citizens. While our argument that territory is a performative effect aligns with relational approaches in human geography, we contend that such performances should be explored through a more fine-grained lens to capture how subjects, rights, and territory are enacted simultaneously. Similarly, while the perspective of noncitizenship as a multifaceted and denationalized category is commonplace in the literature, our analysis of registration highlighted the distinct spatial implications of noncitizen production—demonstrating how territories are brought into being as dynamic geometries that shape mobilities.

Lastly, we have also proposed a comparative research agenda to the study of registration by juxtaposing the Eurodac/Dublin infrastructure, the temporary protection infrastructure, and the Hellenic Register of Foreigners. Following Sassen (2006), comparison can be an important exercise in overcoming the nation-state as an analytical category but still poses questions about how some of its crucial constituent elements are assembled (i.e., territory, rights, and authority). Thus, comparison is “intrinsically spatial” (Robinson, 2016, 306), provoking questions about what physical or symbolic territorializations allow phenomena or elements to emerge. To do so, we suggested to focus on processes of attachment that enacted a database subject together with specific territorial arrangements. As Deville et al. (2016) propose, practicing comparison in this way “involves not a definitive fixing of the qualities of the world but a ‘holding steady’ just long enough for questions of difference and similarity to come into view” (p. 38). Further research on registration infrastructures could build on such an approach and reveal how forms of belonging shape and are shaped by territorial and political settings.

## Notes

1. Torpey drew from to the German term “Erfassung,” that is, apprehending or laying hold of people.

2. Such approach challenges the liberal concept of citizenship, also criticized by Papadopoulos and Tsianos (2013). However, noncitizens should not be conflated with stateless persons. Especially in the context of the EU, migrants and refuges are citizens that arrive from outside the EU (often undocumented).

3. Given linguistic constraints and the *Processing Citizenship*’s task plan organized as a matrix, some data might have been collected by other researchers employed as collaborators in the Project. The Project’s data management plan, the ethical plan and the legal framework underpinning the Grant Agreement provide for this distributed collection. Other than the authors, no other project collaborators contributed to the analyses reported in this article, nor to its writing, and the authors are solely responsible for it.

4. All quotes in this article were either kept in the original or translated into English by the authors.

5. As of 3 January 2024. Source: UNHRC, see https://data2.unhcr.org/en/situations/ukraine.

6. Estimates have been difficult due to the absence of strict border controls which meant that border crossings could not be counted.

7. As Amelung (2023) illustrates, Eurodac’s recently expanded functions both enforce the legal obligation of EU member states to register data and reidentify travelers on the assumption of a future crime, illegal activity, or terrorist offense. This further normalizes suspicion inscribed through what we call attachment.

8. It mentioned initially 650,000 displaced persons, counted as of 1 March 2022, who “had arrived in the Union from Ukraine through Poland, Slovakia, Hungary and Romania” (EU, 2022, 1) and which, at the time of writing, have risen to over 8 million according to the UNHRC.

9. As Vavoula (2022) recalls, legal grounds have not been in place to register persons of temporary protection in one of the EU’s existing systems such as Eurodac. This meant that any commitments about exchanging data on persons who should enjoy benefits from this protection scheme were left to be vague at best.

10. See the EC’s “10-Point Plan for a stronger European coordination on welcoming people fleeing the war from Ukraine,” https://home-affairs.ec.europa.eu/10-point-plan-stronger-european-coordination-welcoming-people-fleeing-war-ukraine_en.

11. GVS/BIS stands for “Grundversorgungs/Betreuungsinformationssystem.”

12. As per the TPD, these benefits must at a minimum include entry to the labor market or the option for self-employment, access to social housing, basic medical care, essential means of subsistence, educational opportunities for minors, and the possibility of family reunification.

13. To become in legal terms a Ukrainian national residing in Ukraine before 24 February 2022 (EU, 2022, 2).

14. Other “target group” categories that were (in some member states) granted temporary protection were defined as “stateless persons, and nationals of third countries other than Ukraine, who benefited from international protection […] in Ukraine before 24 February 2022” (EU, 2022, 5).

15. European governments decided against applying the TPD’s Article 11, which holds that a state “shall take back a person enjoying temporary protection on its territory, if the said person remains on, or seeks to enter without authorization onto, the territory of another Member State” (EU, 2001, Art. 11; EU, 2022, 3).

16. National member states have made Ukrainian citizenship as the core principle for issuing protection to displaced refuges from the war. This is made very clear by the EC operational guidelines (EC 2022), which suggest enacting a very different European territory for those not registered as temporarily protected: non-Ukrainians or persons that could be otherwise considered as security threat, organized crime, or “illegal immigration risks.” In other words, for those unable to become attached to the infrastructure of temporary protection, the new European corridors remained closed as ever.

17. At the time of writing, this has in some cases already been replaced with a “smart card” that likewise indicates the person’s geographical restriction.

18. It should be noted that the Greek Council for Refugees documents diverging practices between locations in Greece, see https://asylumineurope.org/reports/country/greece/reception-conditions/access-and-forms-reception-conditions/freedom-movement/.
